# Low-Dose Lithium Stabilizes Human Endothelial Barrier by Decreasing MLC Phosphorylation and Universally Augments Cholinergic Vasorelaxation Capacity in a Direct Manner

**DOI:** 10.3389/fphys.2016.00593

**Published:** 2016-12-06

**Authors:** Bert Bosche, Marek Molcanyi, Soham Rej, Thorsten R. Doeppner, Mark Obermann, Daniel J. Müller, Anupam Das, Jürgen Hescheler, R. Loch Macdonald, Thomas Noll, Frauke V. Härtel

**Affiliations:** ^1^Division of Neurosurgery, St. Michael's Hospital, Keenan Research Centre for Biomedical Science and the Li Ka Shing Knowledge Institute of St. Michael's Hospital, Department of Surgery, University of Toronto Toronto, ON, Canada; ^2^Department of Neurology, University Hospital of Essen, University of Duisburg-Essen Essen, Germany; ^3^Institute of Neurophysiology, Medical Faculty, University of Cologne Cologne, Germany; ^4^Department of Neurosurgery, Research Unit for Experimental Neurotraumatology, Medical University Graz Graz, Austria; ^5^Division of Geriatric Psychiatry, Department of Psychiatry, Sunny Brook Health Sciences Centre, University of Toronto Toronto, ON, Canada; ^6^Geri-PARTy Research Group, Department of Psychiatry, Jewish General Hospital, McGill University Montréal, QC, Canada; ^7^Department of Neurology, University of Göttingen Medical School Göttingen, Germany; ^8^Center for Neurology, Asklepios Hospitals Schildautal Seesen, Germany; ^9^Pharmacogenetics Research Clinic, Campbell Family Mental Health Research Institute, Centre for Addiction and Mental Health Toronto, ON, Canada; ^10^Department of Psychiatry, University of Toronto Toronto, ON, Canada; ^11^Medical Faculty Carl Gustav Carus, Institute of Physiology, Technical University of Dresden Dresden, Germany

**Keywords:** bipolar disorder, blood-brain barrier, endothelial barrier, endothelial function, myosin light chain, lithium, stroke, vessel relaxation

## Abstract

Lithium at serum concentrations up to 1 mmol/L has been used in patients suffering from bipolar disorder for decades and has recently been shown to reduce the risk for ischemic stroke in these patients. The risk for stroke and thromboembolism depend not only on cerebral but also on general endothelial function and health; the entire endothelium as an organ is therefore pathophysiologically relevant. Regardless, the knowledge about the direct impact of lithium on endothelial function remains poor. We conducted an experimental study using lithium as pharmacologic pretreatment for murine, porcine and human vascular endothelium. We predominantly investigated endothelial vasorelaxation capacities in addition to human basal and dynamic (thrombin-/PAR-1 receptor agonist-impaired) barrier functioning including myosin light chain (MLC) phosphorylation (MLC-P). Low-dose therapeutic lithium concentrations (0.4 mmol/L) significantly augment the cholinergic endothelium-dependent vasorelaxation capacities of cerebral and thoracic arteries, independently of central and autonomic nerve system influences. Similar concentrations of lithium (0.2–0.4 mmol/L) significantly stabilized the dynamic thrombin-induced and PAR-1 receptor agonist-induced permeability of human endothelium, while even the basal permeability appeared to be stabilized. The lithium-attenuated dynamic permeability was mediated by a reduced endothelial MLC-P known to be followed by a lessening of endothelial cell contraction and paracellular gap formation. The well-known lithium-associated inhibition of inositol monophosphatase/glycogen synthase kinase-3-β signaling-pathways involving intracellular calcium concentrations in neurons seems to similarly occur in endothelial cells, too, but with different down-stream effects such as MLC-P reduction. This is the first study discovering low-dose lithium as a drug directly stabilizing human endothelium and ubiquitously augmenting cholinergic endothelium-mediated vasorelaxation. Our findings have translational and potentially clinical impact on cardiovascular and cerebrovascular disease associated with inflammation explaining why lithium can reduce, e.g., the risk for stroke. However, further clinical studies are warranted.

## Introduction

The mood stabilizer lithium has been successfully used in patients suffering from bipolar disorder for decades. Safe therapeutic concentrations of lithium are typically below 1 mmol/L in these patients (Geddes and Miklowitz, 2013; Yatham et al., 2013; Mohammad and Osser, 2014). In preclinical and clinical research, lithium was recognized for robust neuroprotective effects regarding various pathologic conditions (Vo et al., 2015; Doeppner et al., 2016; Vosahlikova and Svoboda, 2016). Recent studies have also identified protective effects of lithium in cardiovascular and cerebrovascular diseases (Gold et al., 2011; Chiu and Chuang, 2012). This protective effect was highlighted by two recent clinical studies demonstrating that prolonged lithium treatment reduces the risk of ischemic stroke in bipolar disorder patients (Lan et al., 2015), and improves neurological recovery after cortical stroke (Mohammadianinejad et al., 2014). Stroke and thromboembolism risk depend not only on cerebral but also on general endothelial functioning. The entire body's endothelium is therefore relevant for these pathologies. However, the impact of lithium on the endothelium and vasomotor tone and potential underlying mechanisms remain poorly understood. In light of the clinical effectiveness of lithium in stroke, we have recently examined lithium-endothelium interactions (Bosche et al., 2013, 2016). Lithium treatment (Rajkowska, 2000; Lan et al., 2015) may be effective in both ischemic and hemorrhagic stroke, and even traumatic brain injury (Leeds et al., 2014; Gao et al., 2016) by improving disturbances in endothelial functions, such as: vascular or cerebrovascular autoregulation of blood flow, vasorelaxation capacity, and dynamic endothelial barrier permeability (Bosche et al., 2003, 2009, 2010; Gündüz et al., 2003; Butcher et al., 2004; Dohmen et al., 2007; Meisel et al., 2012; Renú et al., 2015; Helbok et al., 2016).

Maintenance of intracellular calcium homeostasis in cells of the vessel wall is a prerequisite for endothelium-mediated control of vascular tone (Förstermann and Münzel, 2006; Rahimzadeh-Rofouyi et al., 2007; Bosche et al., 2009, 2010, 2016) and preservation of the endothelial barrier (Garcia et al., 1995; Bosche et al., 2013; Bosche and Macdonald, 2015), which are both determinants of the physiological endothelial function and vascular health (Yoo and Kim, 2009; Grove et al., 2015). In neurons and glia, but perhaps also in the vascular endothelium, lithium may predominantly interact with two enzymes: inositol monophosphatase (IMPase) and glycogen synthase kinase-3 beta (GSK-3β), both of which control a variety of cellular effectors involving intracellular calcium concentration [Ca^2+^]_i_ (Berridge, 1989, 2014; Garcia et al., 1995; Schäfer et al., 2001; Gould and Manji, 2005; Ryglewski et al., 2007; Munaron and Fiorio Pla, 2009; Trepiccione and Christensen, 2010; Bosche et al., 2013). Taken together, there is accumulating evidence indicating that lithium may have protective effects also on vessel function. On the other hand, conflicting results have been published for the impact of low lithium concentrations on vascular and endothelial functions; then human data are lacking almost completely (Bakken et al., 1992; Afsharimani et al., 2007; Rahimzadeh-Rofouyi et al., 2007; Yoo and Kim, 2009; Bosche et al., 2016). Furthermore, there is surprisingly no human data investigating whether low-dose lithium can actually improve endothelial dynamic barrier functioning. Therefore, our current experimental study fills a gap of knowledge with translational and perhaps clinical implications (Bosche and Macdonald, 2015).

Focusing on the pharmacologic interplay of low therapeutic lithium with murine, porcine and human endothelium, we hypothesized that endothelium-mediated vasomotor function may be ubiquitously improved in different species and different vascular provinces, including the cerebral one. In addition, we assume that endothelial barrier property such as the dynamic barrier of human endothelium may be stabilized and thus protected against imminent failure by low therapeutic lithium concentrations. Verifying these hypotheses may have immediate clinical impact as lithium treatment paradigms might be shifted toward broader indications in the future. To our knowledge, this is the first study proving human dynamic endothelial barrier to be stabilized by a pharmacologic treatment with low therapeutic lithium doses.

## Materials and methods

This experimental study was approved by the University Commission on Animal Experiments with respect to the animal welfare regulations of Germany, in accordance to the European Communities Council Directive and to the National Institutes of Health (NIH) Guidelines. The study were approved by the University Ethics Committee of the Medical Faculty Carl Gustav Carus and conformed to the principles of the “Declaration of Helsinki.” It was conducted under permission EZ 203112005 of the local authorities.

### Murine vessel preparation

The vessel grafts were isolated from murine aortas. Vessel preparation was performed according to a slightly modified method as previously described (Wilbring et al., 2013; Kopaliani et al., 2014; Bosche et al., 2016). In brief, male CD57 mice 10 weeks of age (Charles River Laboratories, Sulzfeld, Germany) were sacrificed by cutting off the upper cervical spinal cord under deep anesthesia. After death, the mice were immediately dissected. The pars thoracalis of the aorta (distal of the aortic arch) from murine aortas were recovered, explanted and directly placed into Tiprotec™ solution only (Dr F. Köhler GmbH, Bensheim, Germany), or supplemented with 0.2 or 0.4 mmol/L lithium chloride or carbonate (Sigma-Aldrich, Taufkirchen, Germany). The vessel grafts were stored at 4°C for 48 h. In addition, some vessel grafts were stored at physiologic 37°C for 6 h only; these vessel grafts were incubated with 100 IU/ml Penicillin and 100 μg/ml Streptomycin (GIBCO Life Technologies Eggenstein, Germany) to avoid contaminations. The osmolarity of the Tiprotec™ solution was 305 mosmol/L and the pH 7.0, respectively. The solution contained a mixture of substances with individual concentrations shown in Table 1.

**Table 1:** **Substances of the tissue protecting solution (Tiprotec™) and their respective concentrations**.

**Substance**	**Concentration**
Alpha-Ketoglutarate	2 mmol/L
Aspartate	5 mmol/L
N-acetyl-histidine	30 mmol/L
Glycine	10 mmol/L
Alanine	5 mmol/L
Tryptophan	2 mmol/L
Sucrose	20 mmol/L
Glucose	10 mmol/L
Chloride	103 mmol/L
Sodium	16 mmol/L
Potassium	93 mmol/L
Magnesium	8 mmol/L
Calcium	50 μmol/L
Deferoxamine	82 μmol/L
LK 614	17 μmol/L

### Porcine cerebral vessel preparation

Cerebral vessels were taken from gyrencephalic porcine brains. The porcine cerebral vessel segments were isolated from the proximal part of the middle cerebral artery (M1 segment) from freshly slaughtered male swine (Sus domesticus, 24–26 weeks of age). Extracted vessels were collected and transported in a storage solution Tiprotec™ at 4°C. Subsequently the isolated cerebral M1 vessel segments were flushed, cut and stored either in Tiprotec™ solution only serving as a control or Tip-rotec™ solution supplemented with 0.4 mmol/L lithium carbonate and stored at 4°C for at least 72 h.

### Choice of specific type of arteria and endothelium

The thoracic aorta and the middle cerebral arteria were chosen as studied vessel types for two reasons. (1) The risk of stroke and in particular the risk of arterial thromboembolism is mainly based on thoracic/cervical and cerebral arteries. (2) The aorta is an elastic type artery containing both the ordinary vascular smooth muscle cells (SMC) and the myointimal SMC in a relatively high number. Furthermore, aortic endothelial cells were used in our previous vessel graft and cell culture studies regarding [Ca^2+^]_i_ measurements after long-term and immediate use of lithium and its influence on the specific type of endothelial cells taken from the aorta (Schäfer et al., 2001; Bosche et al., 2013). Compared to the described aortic vessel type, cerebral arteries show different specific characteristics such as habitually missing the *Windkessel function*, because of having less elastic fiber, less myointimal SMC, and differently responding to certain physiological stimuli. Because of these pathophysiologic reasons, we were particularly interested to study both thoracic/cervical arteries that supply the brain as well as the specialized brain arteries including both specific types of endothelium.

### Isometric force measurement of different vessel types

Vessel function was assessed according to the method of Mulvany and Halpern (1977) as described previously (Wilbring et al., 2013; Kopaliani et al., 2014; Bosche et al., 2016). Briefly, aortic and cerebral vessel grafts (2 mm in length and 0.5–0.6 or 1.2–1.4 mm internal width, respectively) were transferred to carbogen equilibrated phosphate saline solution (PSS; in mmol/L: 119 NaCl, 4.7 KCl, 2.5 CaCl_2_, 1.17 MgSO_4_, 1.18 KH_2_PO_4_, 25 NaHCO_3_, 5.5 glucose, 0.027 EDTA) and equilibrated for 30 min at 37°C and subsequently mounted in a myograph (DMT-610 M, Power Laboratory/400; AD-Instruments, Spechbach, Germany) for isometric force measurements. The DMT tissue bath system 700 MO™ in combination with PowerLab Data Acquisition System™ (AD-Instruments Spechbach, Germany) was used for data acquisition. Data recording was performed with LabChart™ software (AD-Instruments Spechbach, Germany). For maximal responses, vessels were stretched with a resting tension that was equivalent to an intraluminal pressure of 100 mmHg. After an accommodation phase of 10 min, when a steady state tension had been reached, maximal contraction with potassium-enriched PSS solution (124 mmol/L KCl) and/or 10 μmol/L phenylephrine (α1-adrenoceptor agonist) was recorded. After inducing a steady-state preconstriction with 10 μmol/L phenylephrine, concentration-response curves were determined for vessel relaxation with acetylcholine (ACH) and sodium nitroprusside (SNP) to assess endothelium-dependent and/or endothelium-independent relaxations.

### Drugs inducing endothelium-dependent and -independent relaxation responses

We used acetylcholine (Sigma-Aldrich) to stimulate the endothelial nitric oxide (NO) production and thereby provoked an endothelium-dependent vasodilatation. Sodium nitroprusside (Sigma-Aldrich) was applied to induce endothelium-independent vasodilatation by directly decreasing the vascular SMC tone. The vessels grafts were pre-contracted by using phenylephrine (Sigma-Aldrich), which induced a SMC-mediated vasoconstriction.

### Human endothelial cell isolation and cultivation

Human endothelial cells were isolated from umbilical cords and cultured as described previously (Gündüz et al., 2003; Aslam et al., 2010). Briefly, the cells were cultured in PromoCell™ endothelial cell basal medium (PromoCell, Heidelberg, Germany) supplemented with 10% (vol/vol) fetal calf serum, 0.4% (vol/vol) endothelial growth supplement with heparin, 0.1 ng/ml human endothelial growth factor, 1.0 μg/ml hydrocortisone, 1 ng/ml bovine fetal growth factor, and 2% (vol/vol) penicillin/streptomycin in humidified atmosphere at 37°C, 5% CO_2_. Confluent monolayers were trypsinized in phosphate-buffered saline [PBS; composition in mM: 137 NaCl, 2.7 KCl, 1.5 KH_2_PO_4_, and 8.0 Na_2_HPO_4_, at pH 7.4, supplemented with 0.05% (wt/vol) trypsin, and 0.02% (wt/vol) EDTA] and seeded at a density of 7 × 10^4^ cells/cm^2^ on 24 mm round Corning Transwell™ polycarbonate membrane filters (0.4 μm). Four days after seeding, the experiments were performed with confluent monolayers of passage #1.

### Measurement of macromolecule permeability of human endothelium

The macromolecule permeability of endothelial cells was determined by the flux of trypan-blue labeled albumin (60 μM) through the cell monolayer in a two-compartment system separated by a filter membrane as described previously (Noll et al., 1999; Gündüz et al., 2003) This albumin flux to the abluminal chamber was continuously monitored spectrophotometrically (Specord 10; Carl Zeiss). After an equilibration period of 10–15 min thrombin was added at a final concentration of 0.2 U/ml as previously described (Aslam et al., 2010), while control cells received the same volume of solvent. In some experiments, we used (instead of thrombin) the peptide derived from the protease-activated receptor-1 (PAR-1), i.e., TFLLR-NH_2_ (Tocris Bioscience, Bristol, UK)—a selective PAR-1 receptor agonist at a final concentration of 12 μM. On the other hand, comparative pre-experiments showed that mouse microvascular endothelial monolayers did not reach similarly tight permeability values in our culture model such as found for the established and well-optimized human endothelium approach. Thus, human endothelium had priority for our model.

### Quantification of myosin light chain phosphorylation in human endothelial cells

The myosin light chain (MLC) phosphorylation in human endothelial cells was measured by western blot analysis (Aslam et al., 2010). Therefore, cells were harvested in 2x SDS-PAGE sample buffer and separated by 12.5% SDS-PAGE and transferred onto nitrocellulose membranes by semi-dry blotting. Membranes were probed using anti-phospho-MLC-2 (Cell Signaling Technology, Danvers, MA, USA) and anti-actin (Sigma-Aldrich) in Tris-buffered saline with 0.1% (v/v) Tween 20 and 5% (w/v) BSA in a dilution of 1:3000 and 1:5000, respectively. Respective secondary HRP-conjugated anti-rabbit and anti-mouse IgG antibodies (Amersham BioSciences Buckinghamshire, UK) were used in a dilution of 1:10:000. Immunoreactivity was detected by Fusion-FX7 (PeqLab, Erlangen, Germany) with enhanced chemiluminescence and quantified by densitometric analysis by using Quantity One software (Bio-Rad, Munich, Germany). MLC phosphorylation was expressed in relation to the intracellular amount of actin.

### Statistical analyses

Results are expressed as mean ± SEM. Confident intervals (CI) are additionally given in some experiments. Regarding the number of groups, intergroup differences were analyzed using independent-sample *t*-test according to Student, or one-way analysis of variance (ANOVA) with *post-hoc* Bonferroni correction for multiple comparisons of three or more groups. The general linear model for repeated measures with *post-hoc* Bonferroni correction for multiple comparisons was performed to analyze both within subject factors over time and between group factors. *P* < 0.05 was considered to be significant. Data analyses were performed using IBM SPSS (IBM, Chicago, IL, USA).

## Results

### Low therapeutic lithium concentrations augment endothelium-dependent but not endothelium–independent relaxation of mouse thoracic arteries

**Figure 1:**
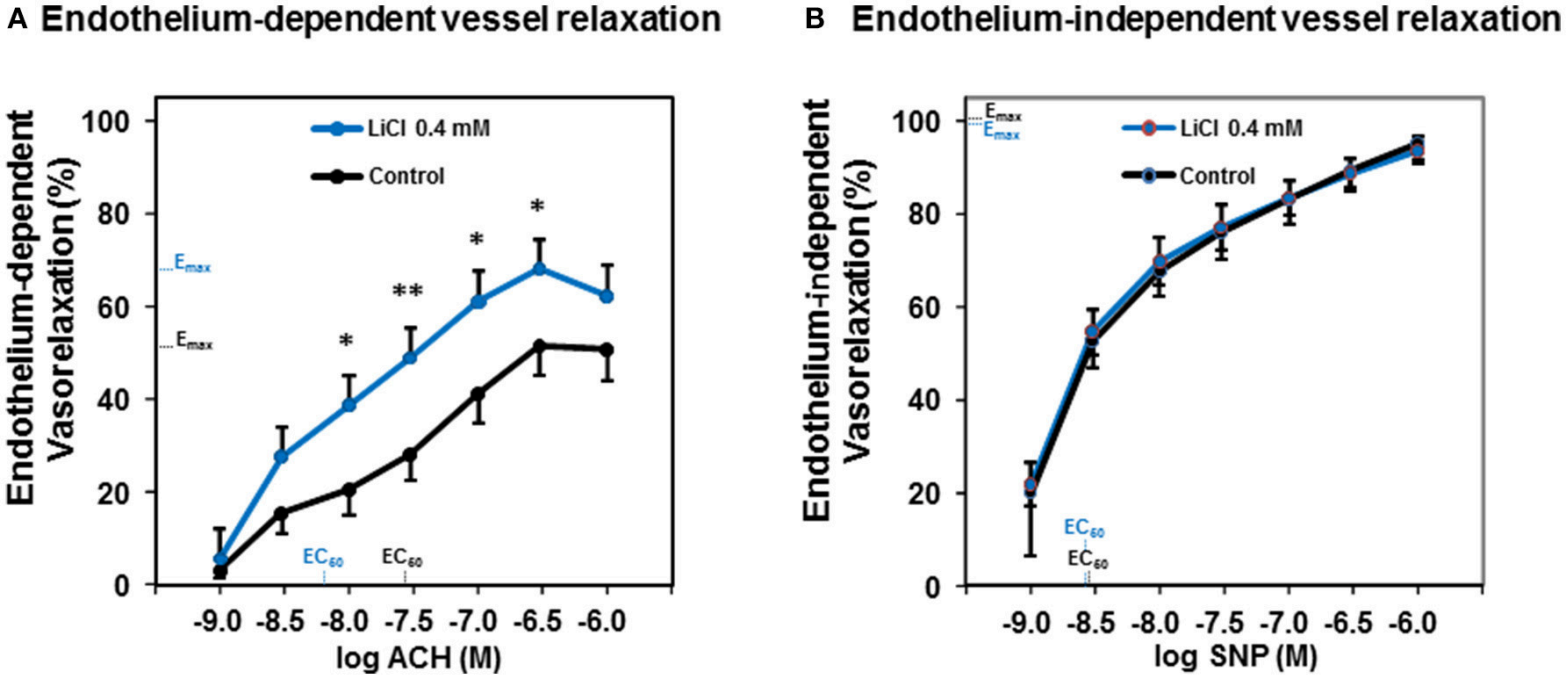
**Dose-dependent acetylcholine- and sodium nitroprusside-induced vessel relaxation and the influence of lithium on those relaxation capacities. (A)** The lithium chloride pre-treated (0.4 mmol/L) murine thoracic arteries showed a significantly improved endothelium-dependent relaxation compared to control at an acetylcholine concentration range from 10^−8^ to 10^−6.5^ M. **(B)** Whereas, the lithium treatment did not significantly alter the endothelium-independent vessel relaxation at any sodium nitroprusside concentration tested. Data are shown in percent of the maximal vessel relaxation and expressed as mean ± SEM of *n* = 4–6 vessels per group of independent preparations, ^*^P < 0.05, ^**^P < 0.01 compared to control, respectively. E_max_, maximal possible effect; EC_50_, half maximal effective concentration for the ACH respectively SNP.

To test whether a lithium treatment at low therapeutic concentrations improves the vessel relaxation capacity, we used murine aortal vessels and ACH as an endothelium-dependent vasodilator besides SNP as an endothelium-independent one. Figure 1A shows that a pharmacologic treatment with 0.4 mmol/L lithium chloride significantly augmented the endothelium-dependent vessel relaxation capacity of ACH in the dose range from 10^−8^ to 10^−6.5^ mol/L compared to control. After this lithium chloride treatment the maximal ACH-induced vessel relaxation was found at an ACH concentration of 10^−6.5^ mol/L (Figure 1A). Investigations of the endothelium-independent vessel relaxation capacity using SNP are illustrated in Figure 1B. The treatment of vessels with 0.4 mmol/L lithium chloride did not significantly alter the endothelium-independent relaxation capacity compared to controls at any SNP concentration tested. Both dose response curves were found nearly congruent (Figure 1B).

**Figure 2:**
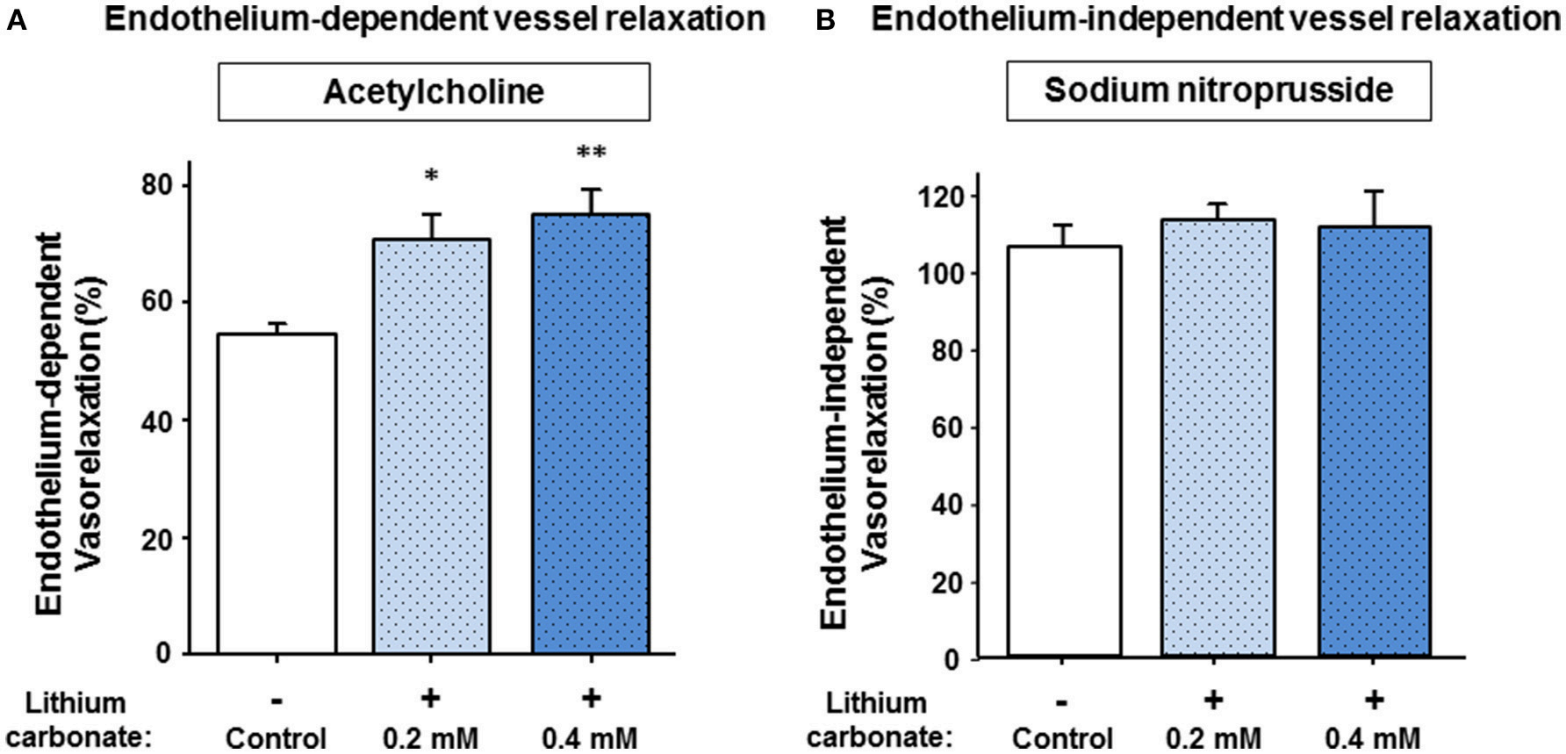
**Acetylcholine- and sodium nitroprusside-induced vessel relaxation and the influence of lithium pre-treatment on relaxation capacities. (A)** The lithium carbonate pre-treated (0.2 and 0.4 mmol/L) murine vessels showed significantly improved maximal endothelium-dependent vessel relaxation compared to control. **(B)** Whereas, lithium treatment did not significantly change maximal endothelium-independent vessel relaxation capacities. Data are shown as percent of the maximal vessel relaxation and expressed as mean ± SEM of *n* = 4–5 vessels per group of independent preparations, ^*^P = 0.018, ^**^P = 0.004 compared to control, respectively.

Since lithium carbonate is predominantly used for clinical treatment, we then tested whether lithium carbonate may also improve the vessel relaxation capacity after stimulating with ACH and/or SNP, respectively. Figure 2A illustrates that either a treatment with 0.2 or 0.4 mmol/L lithium carbonate significantly augmented the maximal ACH-induced vessel relaxation capacity compared to control. Thereby, the later lithium carbonate concentration (0.4 mmol/L) most sufficiently increased the relaxation capacity leading to a highly significant difference compared control (Figure 2A). Figure 2B reveals that we found neither for 0.2 nor for 0.4 mmol/L lithium carbonate a significant difference of the maximal SNP-mediated (endothelium-independent) vessel relaxation between lithium treated vessels and control. These experiments (compare Figure 2A and Figure 2B) were partly repeated following a modified protocol using a lithium pre-treatment at 37°C for 6 h. We found similar results; lithium carbonate (0.4 mmol/L) significantly increased the maximal ACH-induced vessel relaxation compared to control (82.36 ± 2.36% vs. 52.94 ± 5.52%, *n* = 4–5 per group, *P* = 0.003). Whereas, the SNP-induced vessel relaxation was not significantly altered by lithium carbonate (105.09 ± 4.08% vs. 102.63 ± 1.04%, *n* = 5 per group, *P* = 0.583, n.s.); similar (non-significantly altered) results were found when submaximal SNP concentrations (e.g., 10^−8.5^ mM) were used for vasodilatation with or without 0.4 mmol/L lithium carbonate pre-treatment (80.15 ± 6.45% vs. 81.01 ± 13.82%, *n* = 5 per group, *P* = 0.902, n.s).

### Low therapeutic lithium concentrations improve endothelium-dependent relaxation of cerebral arteries

**Figure 3:**
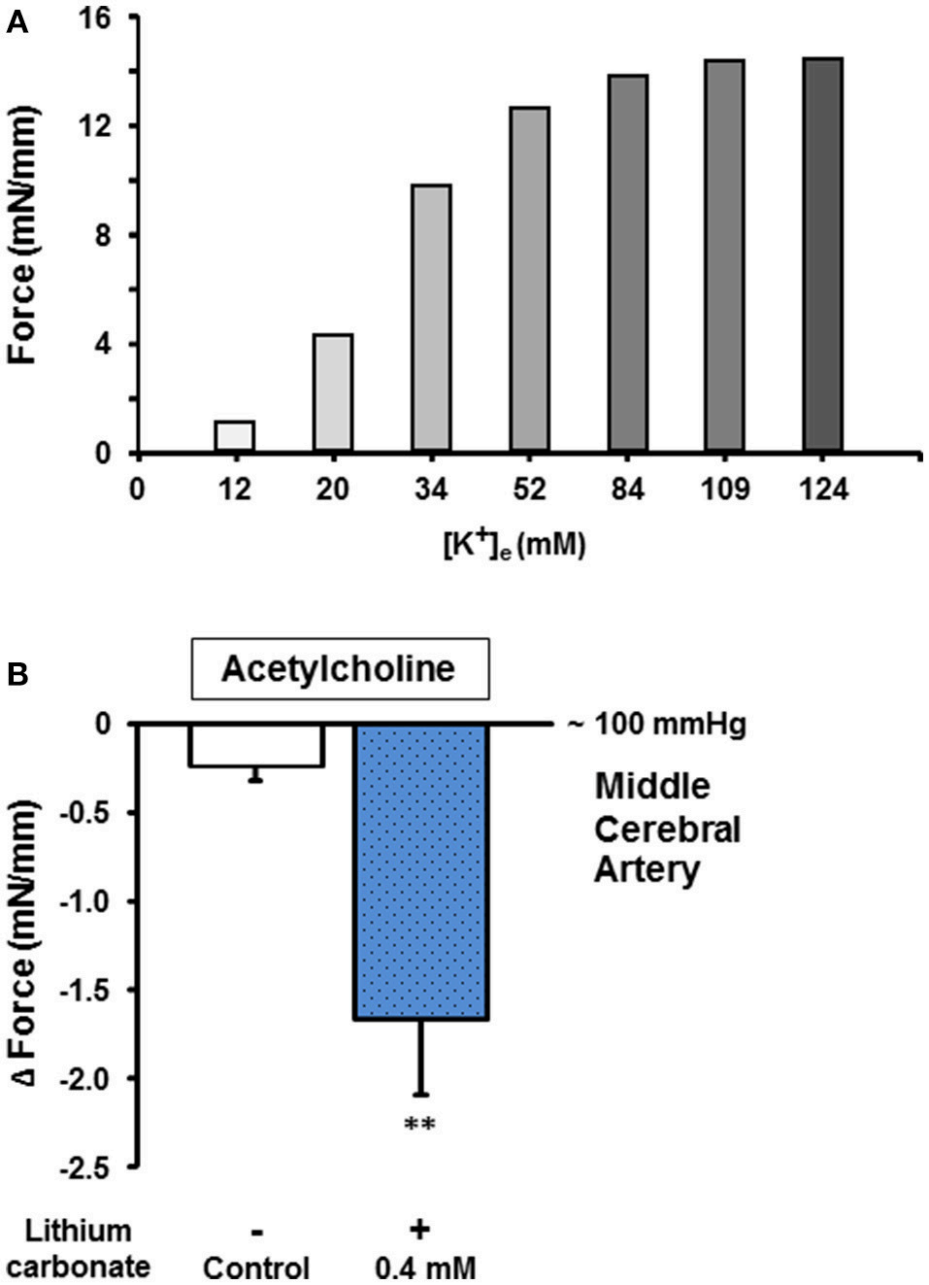
**Endothelium-dependent cholinergic vessel function and the influence of lithium carbonate on the relaxation capacity of cerebral arteries. (A)** Representative pre-assessment of physiologic vessel functions (e.g., potassium-induced constriction force [mN/mm]) for further relaxation capacity measurements. **(B)** The lithium carbonate (0.4 mmol/L) pre-treated porcine middle cerebral arteries showed significantly improved endothelium-dependent vessel relaxation compared to control. Data are shown as Δ mN/mm and expressed as mean ± SEM of *n* = 3–4 vessels per group of independent preparations, ^**^P = 0.006 compared to control.

To test whether low lithium concentrations also augment the endothelium-dependent relaxation capacity of cerebral vessels from gyrencephalic brains, we performed another set of experiments using porcine M1 segments of the middle cerebral arteries (MCA) and lithium carbonate treatment. Cerebral vessels of good to excellent functions (e.g., constriction force ≥8 mN/mm for 34 mM [K^+^]_e_, a representative example is given in Figure 3A) were used for these lithium pretreated ACH-induced vasorelaxation experiments. Figure 3B illustrates that 0.4 mmol/L lithium carbonate significantly augmented the endothelium-dependent cerebral vessel relaxation in response to 10^−6.5^ mol/L ACH (at a mechanical pre-dilatation/constriction equal to a vessel lumen pressure of 100 mmHg) compared to control. These findings of porcine cerebral and thoracic vessels indicated lithium (at low therapeutic concentrations) as an agent equally augmenting endothelium-mediated relaxation capacities of different vascular provinces and species in a direct manner (i.e., not via central and autonomic nervous system-associated routes).

### Low therapeutic lithium concentrations appear to reduce resting human endothelial permeability

**Figure 4:**
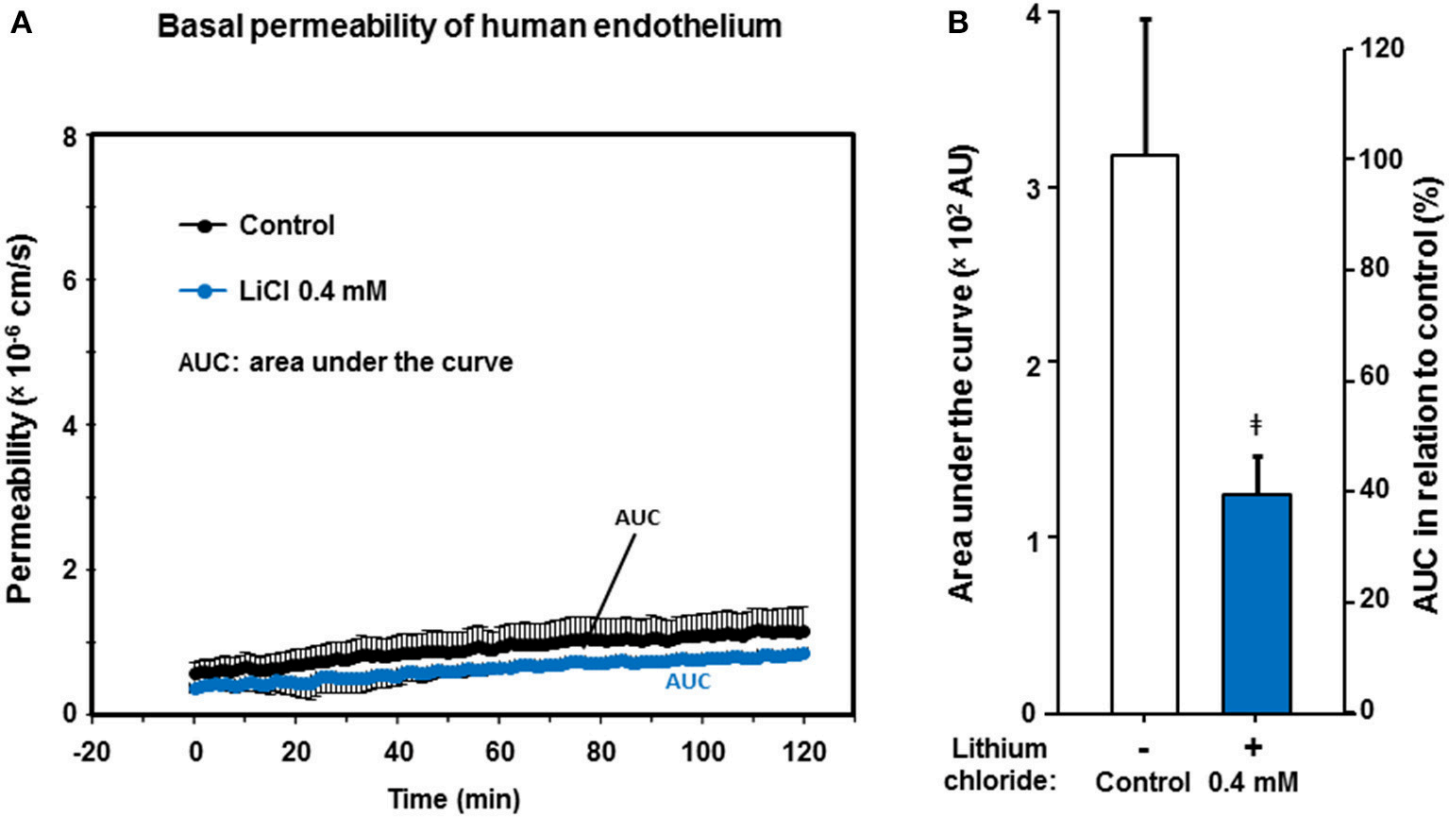
**Effect of lithium at low therapeutic concentrations on the basal permeability of human endothelium. Confluent human endothelial monolayers were exposed to different concentrations of lithium for 48 h. (A)** Basal albumin permeability is shown after pre-treating with Tiprotec™ solution in absence of lithium as control (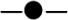) or with the same solution containing 0.4 mmol/L lithium (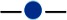). Over the entire observation period, the lithium pre-treated endothelial monolayers showed a lower permeability (i.e., a tighter basal barrier function). Differences were reflected by strong statistical trends at certain time points (see Result Section); AUC, areas under the curve. **(B)** By comparing the integrals, i.e., AUC (compare part **A**) of permeability assessments as a single measure for 120 min, the barrier built by the lithium treated human endothelium appears to be tighter compared to control (^‡^*P* = 0.064, not significant [n.s.]). Data are expressed as mean ± SEM of *n* = 4–6 separate experiments per group of independent cell preparations.

The universally positive influence of low lithium concentrations on the endothelium-dependent vessel relaxation capacity prompted us to further investigate, whether other endothelium functions could also be modified or improved by pharmacological lithium treatment at low therapeutic concentrations. Translationally, we were most interested in human endothelial functions such as endothelium permeability/impermeability. Therefore, we scrutinized the basal permeability of human endothelial monolayers (passage #1), but also the dynamic hyper-permeability induced by thrombin (see below). We continuously accessed the albumin turnover of the monolayers in a resting state that were treated with 0 (control) and 0.4 mmol/L lithium. Over the entire observation period of 120 min, the basal permeability of lithium treated human endothelial monolayers was lower compared to control (Figure 4A). This was, however, only reflected by strong statistical trends comparing the values (e.g., after 40 min, *n* = 4–6 per group, *P* = 0.076) or the integrals, i.e., areas under the curve of permeability to control (AUC, *n* = 4–6 per group, *P* = 0.064, n.s., Figure 4B).

### Low therapeutic lithium concentrations significantly abolish human dynamic endothelial hyper-permeability

**Figure 5:**
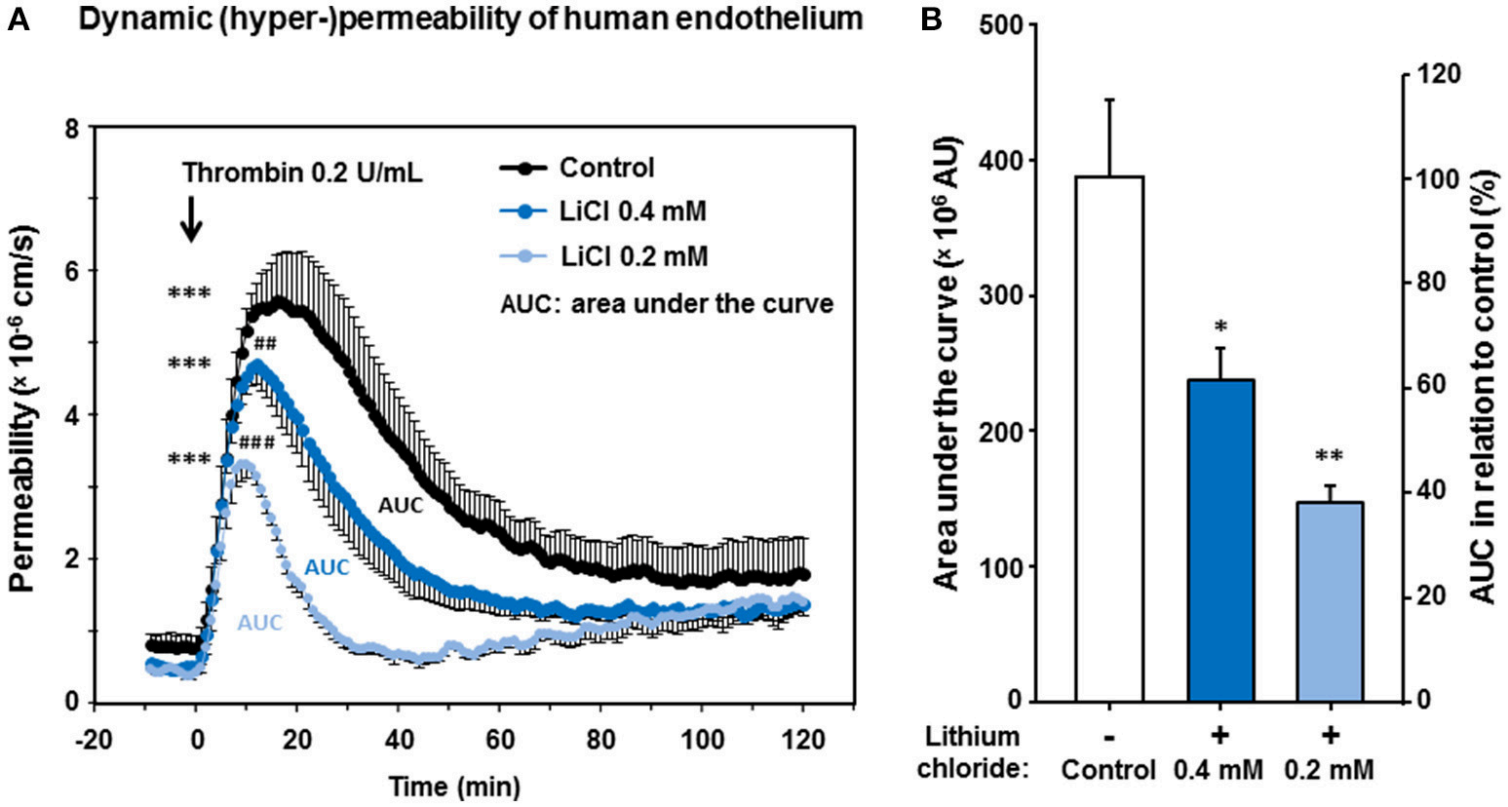
**Effect of lithium at low therapeutic concentrations on the dynamic, thrombin-induced hyper-permeability of human endothelium**. Confluent human endothelial monolayers were exposed to lithium for 48 h. **(A)** Basal and dynamic, thrombin-induced hyper-permeability after pretreating with Tiprotec™ solution in absence of lithium as a control (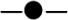) or with the same solution containing 0.4 (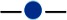) or 0.2 mmol/L lithium (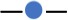). Thrombin induced a highly significant increase in permeability of control and both lithium treated groups (all, ^***^P <0.001 compared to their basal permeability, respectively). Before addition of thrombin the basal albumin permeability of 0.4 and 0.2 mmol/L lithium treated human monolayers was slightly but non-significantly lower compared to control (*P* = 0.095 and *P* = 0.070, n.s., respectively). Both types of lithium treated human endothelium showed a significantly lower dynamic thrombin-induced hyper-permeability compared to control (^*##*^*P* = 0.004 and ^*###*^*P* <0.001, respectively; see also Table 3 for further details). **(B)** Consistently, the dynamic hyper-permeability of lithium treated endothelium (0.4 and 0.2 mmol/L, integrals/AUC, compare part **A**) was significantly lower compared to control (^*^P = 0.019 and ^**^P = 0.003, respectively). Data are expressed as mean ± SEM of *n* = 4–6 separate experiments per group of independent cell preparations.

Since thrombin plays a relevant pathophysiologic role for the endothelial barrier failure or impairment (Coughlin, 2000), e.g., during and after cerebral ischemia and hemorrhages (Stokum et al., 2016), we investigated the impact of lithium treatment on the thrombin-induced hyper-permeability of human endothelium. Regarding the basal permeability before thrombin addition, we again found similarly strong trends between lithium 0.2, respectively, 0.4 mmol/L treated endothelium and control [0.4156 ± 0.0253, respectively 0.5189 ± 0.0714 vs. 0.8315 ± 0.1491 (× 10^6^ cm/s), *n* = 4–6 per group, *P* = 0.070 and *P* = 0.095, both n.s.]. Figure 5A illustrates that thrombin significantly increased the endothelial permeability of all groups in a transient way (all, *P* < 0.001, respectively, Table 2). More importantly, Figure 5 shows that the treatment with lithium chloride (0.4 and 0.2 mmol/L) significantly reduced the permeability/hyper-permeability (*P* = 0.004 and *P* < 0.001, respectively); Table 3 summarizes mean differences with the respective statistics in detail. Correspondingly, hyper-permeability expressed as AUC was significantly lower in human endothelium treated with 0.4 and 0.2 mmol/L lithium compared to control (AUC, *n* = 4–6 per group, *P* = 0.019 and *P* = 0.003, respectively, Figure 5B).

**Table 2:** **Permeability increase (basal to peak) of human endothelium after treatment with different lithium concentrations**.

**Group**	**Mean increase (10^6^ × cm/s)**	**SEM**	***P*-value**
Control	5.3962	±0.4043	<0.001
Lithium 0.2 mmol/L	2.9419	±0.1747	<0.001
Lithium 0.4 mmol/L	3.9716	±0.3881	<0.001

**Table 3:** **Comparison of the dynamic thrombin-induced hyper-permeability of groups of human endothelium treated with or without lithium**.

**Group comparison**		**Mean difference (10^6^ × cm/s)**	**SEM**	***P*-value**	**95% CI**	
					**LB**	**UB**
Control	Lithium 0.2 mmol/L	1.6430	±0.3351	<0.001	0.7533	2.5327
Control	Lithium 0.4 mmol/L	1.0248	±0.2681	0.004	0.3130	1.7366
Lithium 0.4 mmol/L	Lithium 0.2 mmol/L	0.6182	±0.0307	0.181	−0.1972	11.4337

*CI, confidence interval; LB, lower bound; UB, upper bound*.

**Figure 6:**
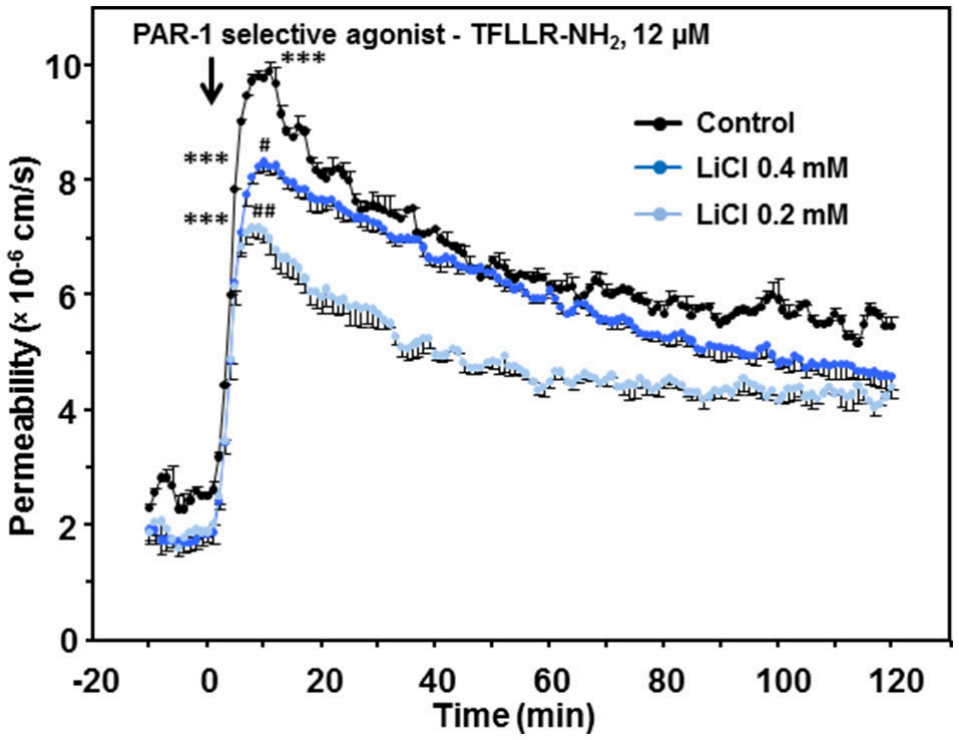
**Effect of lithium at low therapeutic concentrations on the dynamic, TFLLR-NH_**2**_-induced hyper-permeability of human endothelium**. TFLLR-NH_2_ is an oligopeptide (Thr-Phe-Leu-Leu-Arg-NH_2_), which acts as a PAR-1 selective agonist. Confluent human endothelial monolayers were exposed to different concentrations of lithium for 48 h. Basal and dynamic TFLLR-NH_2_-induced hyper-permeability after pretreating with Tiprotec™ solution in absence of lithium as a control (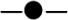) or with the same solution containing 0.4 (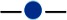) or 0.2 mmol/L lithium (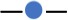). TFLLR-NH_2_ induced a highly significant increase in permeability of control and both lithium treated groups (all, ^***^P < 0.001 compared to their basal permeability, respectively). Both types of lithium treated human endothelium revealed a significantly lower dynamic TFLLR-NH_2_-induced hyper-permeability compared to control (^#^*P* = 0.031 and ^*##*^*P* = 0.001, respectively). Data are expressed as mean ± SEM of *n* = 3 separate experiments per group of independent cell preparations.

To investigate the hypothesis whether the lithium-attenuated dynamic hyper-permeability of human endothelium was mediated by an involvement of the receptor PAR-1 and downstream signaling, we repeated the experiments (compare Figure 5A) using the selective PAR-1 receptor agonist TFLLR-NH_2_. Lithium chloride (0.2 and 0.4 mmol/L) likewise significantly abolished the TFLLR-NH_2_-induced hyper-permeability (Figure 6).

### Lithium-attenuated human dynamic endothelial permeability is mediated by a reduced endothelial myosin light chain phosphorylation

**Figure 7:**
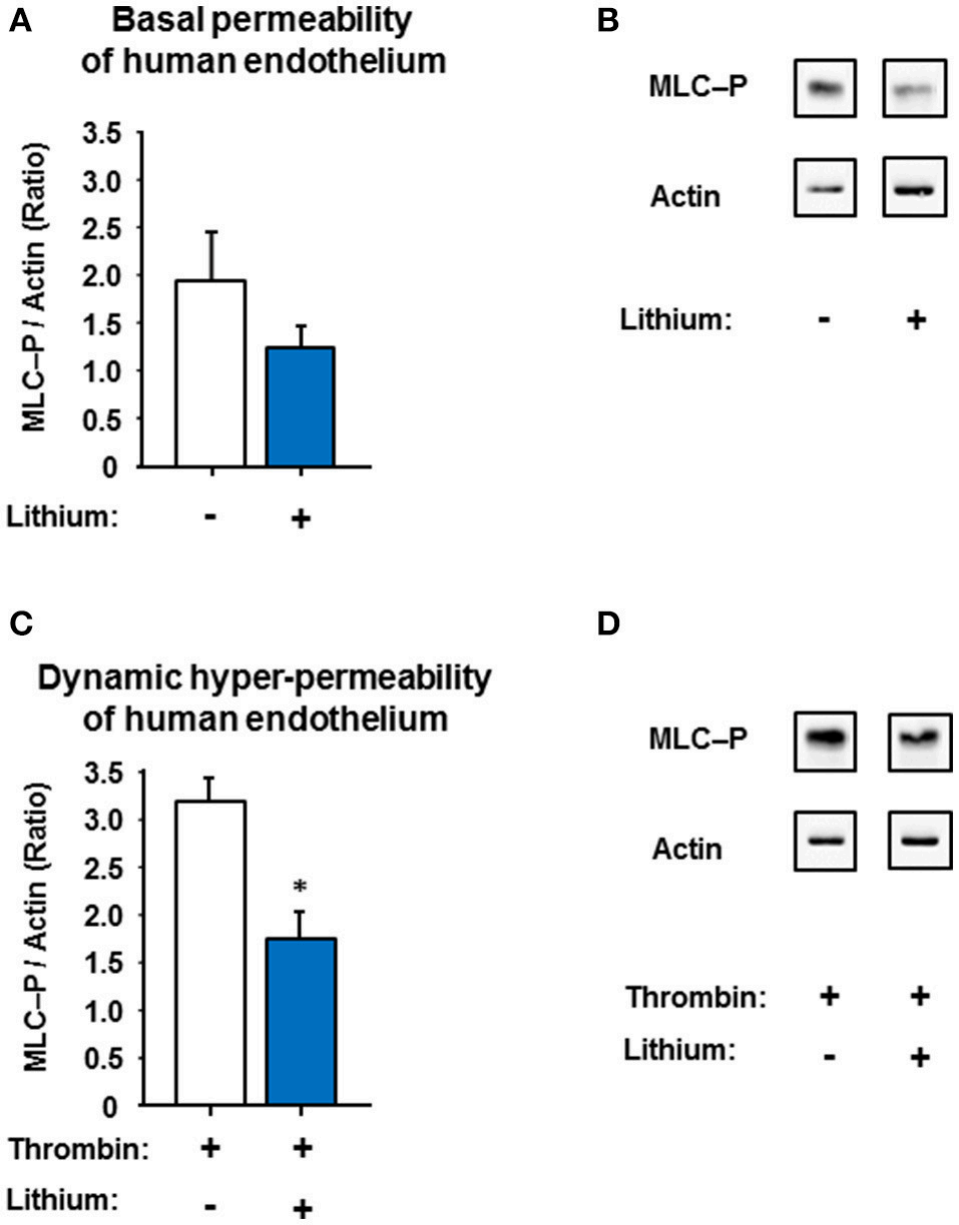
**Effect of low-dose lithium with or without thrombin on the myosin light chain phosphorylation (MLC-P) of human endothelium**. Confluent human endothelial monolayers were exposed to different concentrations of lithium: No lithium (control) and 0.4 mmol/L lithium chloride (compare Figure 4 and Figure 5). **(A)** The basal endothelial MLC-P was somewhat reduced in the human endothelium treated with 0.4 mmol/L lithium chloride, however, in a non-significant way (*P* = 0.275, n.s.). **(B)** Representative western blots under basal permeability conditions with or without lithium, compare part **A**. **(C)** Thrombin (0.2 U/ml) led to an increase in MLC-P of human endothelium, which is known to intensify the endothelial cell contraction with subsequent inter-endothelial gap formation. The thrombin-induced MLC-P increase was significantly abolished in the low-dose lithium (0.4 mmol/l) treated endothelium 5 min after addition of thrombin (^*^P = 0.014 compared to control). **(D)** Representative western blots under dynamic hyper-permeable conditions with or without lithium, compare part **C**. Data from part **A,B** are shown as ratios of the MLC-P/actin and expressed as mean ± SEM of *n* = 3 separate experiments per group.

The endothelial MLC phosphorylation is regulated by the protein kinase C and chiefly controls the contractile apparatus of endothelial cells (Garcia et al., 1995). Due to this phosphorylation the active contractile apparatus [Ca^2+^]_i_-dependently develops small paracellular gaps and thus hyper-permeability (Aslam et al., 2010). Lithium is known to inhibit the inositol monophosphatase/glycogen synthase kinase-3-β signaling-pathways including [Ca^2+^]_i_ in neurons and perhaps other cells (Berridge, 1989, 2014; de Sousa et al., 2015; Bosche et al., 2016). These down-steam pathways may affect MLC phosphorylation; we therefore studied the MLC phosphorylation (with or without lithium) as possible link to endothelial permeability. Figure 7 demonstrates that a prolonged lithium treatment significantly reduced the endothelial intracellular MLC phosphorylation during thrombin-induced hyper-permeable conditions compared to control [1.75 ± 0.26 vs. 3.20 ± 0.23 (ratio of MLC-P/actin) *n* = 3 per group, *P* = 0.014] suggesting a potential mechanism of the lithium-attenuated dynamic permeability of human endothelium.

## Discussion

We conducted an experimental study using lithium as a pharmacologic treatment for murine, porcine and human vascular endothelium with three interrelated goals. First, we wanted to clarify whether low concentrations of lithium help support endothelium-dependent vessel relaxation, since conflicting results had previously been published (Bakken et al., 1992; Afsharimani et al., 2007; Rahimzadeh-Rofouyi et al., 2007; Bosche et al., 2016). Second, we wanted to assess whether low concentrations of lithium carbonate, as a commonly used drug in bipolar disorder and other psychiatric/neurological conditions (Geddes and Miklowitz, 2013; Yatham et al., 2013), correspondingly augment endothelium-dependent thoracic and cerebral vessel relaxation capacity. Third and most important, we aimed to find similar evidence as to whether low lithium concentrations can improve other endothelial functions such as the basal and dynamic permeability (Coughlin, 2000; Gündüz et al., 2003; Aslam et al., 2010; Bosche et al., 2013) particularly of human endothelium (Gündüz et al., 2015). Moreover, we wanted to provide some first hints for a mechanistic explanation of the findings; and modified MLC-P appeared to be a possible candidate.

It is worth to mention that models with denervated vessels were performed to investigate the isolated vessel reaction in direct response to different concentrations of a pharmacologic lithium treatment independently of the influence of lithium on the central and hence the vegetative nerve system including its remote control of the vessel tones.

The data presented here suggested that lithium improved and stabilized endothelium-dependent vascular relaxation capacity and the human endothelial dynamic barrier, respectively. The latter represents a unique finding for human endothelium. After pharmacologic treatment with lithium carbonate at low therapeutic concentrations (up to 0.4 mmol/L), arterial relaxation capacities were significantly improved in different vascular provinces. This means that the improvement was similarly mediated through both aortal and cerebral endothelium. An endothelium-independent mechanism was not involved, in concordance with previous reports showing that removal of the endothelium hindered the lithium-augmented vasorelaxation (Bosche et al., 2016). Moreover, the endothelium-independent NO donor effect (induced by SNP) remained unaltered by lithium again shifting endothelium as a lithium target into focus. The findings of lithium carbonate also originated from two different vertebrate species (mouse and swine) and additionally different vascular provinces suggesting a general rather than a locally circumscriptive endothelial characteristic. We then followed up those experiments, by investigating endothelium isolated from human vessels. The impact of low therapeutic lithium on dynamic endothelial barrier functioning was directly measured for human endothelium and represented a novel finding determining lithium to significantly stabilize endothelial barrier. These findings underline the concept of lithium being a promising approach of targeting human endothelium for treating (or at least positively influencing) vascular and cerebrovascular dysfunctions such as impaired autoregulation and endothelial barrier breakdown, as found after cerebral ischemia (Bosche et al., 2003; Dohmen et al., 2007; Wijdicks et al., 2014) or hemorrhagic stroke such as subarachnoid hemorrhage (Bosche et al., 2009, 2010; Urday et al., 2015). Predominantly, post-ischemic malignant brain edema after hemispheric stroke (Hacke et al., 1996; Bosche et al., 2003) may represent a potential field (Wijdicks et al., 2014) for a pharmacologic treatment with lithium. However, further studies and particularly clinical investigations will be required to provide more definitive conclusions.

The question arises as to how lithium improves endothelial functioning. Similarly to neurons and glia, lithium also intracellularly interacts with IMPase and GSK-3β in vascular endothelium subsequently altering IP_3_, cAMP and thus [Ca^2+^]_i_ (Berridge, 1989, 2014; Schäfer et al., 2001; Gould and Manji, 2005; Ryglewski et al., 2007; Munaron and Fiorio Pla, 2009; Trepiccione and Christensen, 2010; Bosche et al., 2013). In endothelial cells, lithium prevents the discharge of calcium from endogenous storage by inhibition of the inositol trisphosphate (IP_3_)-sensitive calcium channels of the endothelial endoplasmic reticulum (ER), thus counteracting cells stress-induced cytosolic calcium increase and conferring lithium an endothelial cytoprotective potential (Schäfer et al., 2001; Bosche et al., 2013). Functionally, maintenance of [Ca^2+^]_i_ homeostasis at low-dose lithium may manifest as modified endothelium-mediated vasodilation (Förstermann and Münzel, 2006; Rahimzadeh-Rofouyi et al., 2007; Bosche et al., 2016) but also as preserved dynamic endothelial barrier function. Besides the effect of lithium on IP_3_-sensitive [Ca^2+^]_i_ (Berridge, 1989) particularly in endothelial cells previously reported by our group (Schäfer et al., 2001; Bosche et al., 2013), nitric oxide (Bosche et al., 2016), and MLC phosphorylation (Aslam et al., 2010) may serve as downstream targets mediating vasorelaxation and endothelial contraction inducing hyper-permeability, respectively (for review see Stokum et al., 2016). In endothelium, the MLC-P is protein kinase C and [Ca^2+^]_i_/calmodulin-dependent (Garcia et al., 1995; Aslam et al., 2010). By identifying the reduced endothelial MLC phosphorylation after prolonged low-dose lithium treatment, we found a mechanistic explanation for the lithium-attenuated endothelial hyper-permeability and slightly reduced basal permeability. Characterizing the detailed endothelial mechanisms should be the next step for our future research perhaps additionally in an *in-vivo* model. If MLC-P may also be influenced by lithium in vascular SMCs is perhaps likely but unclear, yet, and thus requires also further research.

In light of our current findings, lithium at low therapeutic concentrations functionally represented a universal endothelium protective agent, as reported by others in single species and only one vascular province (Bakken et al., 1992; Afsharimani et al., 2007; Rahimzadeh-Rofouyi et al., 2007). The last of these studies, e.g., found that low lithium concentrations (0.5 mmol/L) reduced and higher ones (2 mmol/L) improved ACH-induced mesenteric vascular bed relaxation, which is partly at odds with our results, perhaps because of the mesenteric vessel type used (Rahimzadeh-Rofouyi et al., 2007). We investigated thoracic and middle cerebral arteries. Relaxation of cerebral vessels during and after an ischemia/reperfusion leads to collateral cerebral blood flow, and thus characterizes an intrinsic strategy of the cerebral vasculature to protect neuroglial structures but also vasculature including endothelium against ischemic injury (Heiss et al., 2001), likewise reported for the heart (Koerselman et al., 2003; Meier et al., 2013). Indeed, cerebral collateral status and sufficiently enlarged calibers of collateral arteries have recently been identified as most relevant for final infarct volume, vasogenic edema formation (with subsequent midline shift), and hence patient outcome (Volny et al., 2016; van den Wijngaard et al., 2016; van der Hoeven et al., 2016). Therefore, patients at risk for stroke with unfortunate collateral status (thus portending poor outcome) could particularly profit from a lithium treatment at low concentrations via a generally improved endothelium-dependent vessel relaxation capacity. This might be speculative, but on the other hand, the lithium-augmented cerebrovascular relaxation capacity may party explain, why continuous lithium treatment can reduce the risk for stroke (Lan et al., 2015) or may improve neurologic recovery after cortical stroke (Mohammadianinejad et al., 2014) potentially caused by various beneficiary effects on neurons (Doeppner et al., 2016; Vosahlikova and Svoboda, 2016), or platelets (Barry et al., 2003) including the direct ones on vascular and cerebrovascular endothelium (Afsharimani et al., 2007; Rahimzadeh-Rofouyi et al., 2007; Bosche et al., 2013, 2016), as presented here. Directly or secondarily impaired endothelial barrier after ischemia and hemorrhages followed by vasogenic edema formation (Stokum et al., 2016) were known to be highly relevant for clinical outcome of various types of stroke (Hacke et al., 1996; Bosche et al., 2003; Macdonald, 2014; Wijdicks et al., 2014; Urday et al., 2015). Therefore, those patients may clinically benefit from a lithium-stabilized, MLC-mediated dynamic endothelial barrier with subsequently reduced vasogenic edema formation. However, additional research is warranted, which will help to better understand the complex phenomenon of lithium-strengthened endothelial barrier and augmented cerbrovascualar relaxation capacities including the potential benefit for stroke patients.

Three limitation of our study deserve mentioning. First, the endothelial intracellular lithium concentration was not directly measured, e.g., by using lithium NMR spectroscopy methods (Fonseca et al., 2004), in our study. On the other hand, serum levels of lithium and not intracellular concentrations are clinically relevant and used for lithium therapy monitoring. Second, a complete mechanistic explanation for all lithium-associated endothelial findings is not yet given in this paper. The known effects of lithium on neurons, particularly on neuronal GSK-3β/IMPase pathways are a matter of extensive research over decades; however, exploring the direct lithium-endothelium interaction has just recently started. Thus, our knowledge is still limited and further research is needed and planed in this field. Third, an ideal model for human endothelial barrier respectively blood-brain barrier does not yet exist (Helms et al., 2016). For investigating (fairly tight) endothelial permeability, we used human endothelial cells of passage #1, aiming to avoid culture effects due to higher passages. Using human cerebral endothelial cells might further improve our particular knowledge about lithium in cerebrovascular diseases. However, commercially available human endothelial cell lines are immortalized and hence of higher passages with many pitfalls restricting translations to the *in-vivo* situation. Hence our approach represents a compromise minimizing some but not all methodologic drawbacks.

In conclusion, a low-dose therapeutic concentration of the mood stabilizer lithium directly stabilizes the human endothelial barrier by reducing MLC phosphorylation weakening the endothelial contractile machinery and thus avoiding paracellular gap formation. Moreover, low-dose lithium augments endothelium-dependent thoracic and cerebral vasorelaxation capacity. These findings of improved endothelial functions could partly explain why long-term lithium treatment reduces the risk for ischemic stroke in patients who receive lithium. Therefore, our results may open a gate for novel lithium indications potentially for patients suffering primarily from cardiovascular and cerebrovascular diseases with impending or already impaired endothelial functions. However, further translational research and clinical studies are warranted.

## Author contributions

All listed authors substantially contributed to this work. BB, MM, FH, and TN designed the study. BB, MM, and FH performed the experiments and acquired the data, which BB, MM, SR, JH, RM, AD, TN, and FH analyzed. BB, SR, TD, MO, DM, TN, and FH wrote the manuscript; all authors reviewed, critically revised, and approved it for final publication. TN and FH contributed equally to this work.

### Conflict of interest statement

BB got a travel grant and a speaker honorary from CSL Behring, Germany. RM is chief scientific officer of Edge Therapeutics, Inc. BB is a member of the scientific advisory board of Edge Therapeutics. BB, RM, and TN got material support of CSL Behring, Germany, and Canada. MO received travel support, and/or speaker honoraria from Biogen Idec, Novartis, Sanofi-Aventis, Genzyme, Pfizer, Teva, and Heel. The other authors declare that the research was conducted in the absence of any commercial or financial relationships that could be construed as a potential conflict of interest.
