# Erratum: Low-Dose Lithium Stabilizes Human Endothelial Barrier by Decreasing MLC Phosphorylation and Universally Augments Cholinergic Vasorelaxation Capacity in a Direct Manner

**DOI:** 10.3389/fphys.2019.00648

**Published:** 2019-05-22

**Authors:** Frontiers Production Office

**Affiliations:** Frontiers Media SA, Lausanne, Switzerland

**Keywords:** bipolar disorder, blood-brain barrier, endothelial barrier, endothelial function, myosin light chain, lithium, stroke, vessel relaxation

Due to a production error it was indicated that a safe therapeutic concentration of lithium was “1 mol/L” in the second sentence of the Introduction. The correct measurement is “1 mmol/L.”

The publisher apologizes for this mistake. The original version of this article has been updated.

